# Advancements in Bio-Based Piezoelectric Composites for Antibacterial Applications

**DOI:** 10.3390/bioengineering13030290

**Published:** 2026-02-28

**Authors:** Ruihua Mu, Xiaoqian Shi, Wenzhuo Chen, Kaige Zhang

**Affiliations:** Xi’an Key Laboratory of Textile Chemical Engineering Auxiliaries, School of Environmental and Chemical Engineering, Xi’an Polytechnic University, Xi’an 710048, China; 18149012271@163.com (X.S.); 19971565119@163.com (K.Z.)

**Keywords:** piezocatalysis, mechanical energy, piezoelectric biomaterials, antibacterial, disinfection

## Abstract

Mechanical energy, a ubiquitous renewable resource, can be effectively harnessed via piezocatalysis to convert physical stimuli into chemical energy for sterilization. As a promising green technology, piezocatalysis employs external mechanical force to physically disrupt bacterial membranes while simultaneously triggering redox reactions to generate bactericidal reactive oxygen species (ROS). Recent advances highlight the superior performance and broad applicability of this technology in the antibacterial domain. This review systematically elucidates the antibacterial mechanisms of piezocatalysis, followed by a comprehensive survey of prevalent piezoelectric biomaterials (e.g., amino acids, cellulose, proteins) and their synthesis strategies. Furthermore, specific applications of these bio-piezoelectric composites in sterilization are consolidated. Finally, we critically assess the primary challenges and outline future developmental trajectories, offering a prospective pathway for next-generation eco-friendly disinfection strategies.

## 1. Introduction

Bacterial infections pose a critical threat to global public health, causing millions of fatalities annually. While antibiotics remain the cornerstone of treatment, their overuse has precipitated the rapid emergence of multidrug-resistant (MDR) strains, creating an urgent crisis [1,2]. Conventional alternatives, such as plasma, ozone, and photocatalytic disinfection, often suffer from limitations including restricted operational radii, generation of carcinogenic by-products, or dependence on light sources [3,4,5]. Consequently, there is an imperative need for novel, efficient, and eco-friendly sterilization methodologies. Piezocatalysis, an innovative technology harnessing ubiquitous mechanical energy via the piezoelectric effect, has emerged as a compelling solution [6]. Unlike traditional approaches, it requires no external chemical agents and operates efficiently under dark conditions, making it a highly sustainable candidate for diverse antibacterial applications.

Specifically, piezocatalysis is activated by material deformation [7,8,9]. This deformation causes an internal polarization phenomena, resulting in the segregation of positive and negative charge centers that accumulate at opposite ends of the material [10]. The polarization enhances the electric field polarization of water, facilitating the interaction between the charges at both ends of the water and the material, ultimately resulting in the generation of hydroxyl groups [11]. Free radicals and other reactive species are employed to eradicate bacteria. Moreover, the mechanical stress exerted during piezoelectric catalysis, such as ultrasonic waves and water shear force, might directly compromise the bacterial cell membrane, indirectly resulting in bacterial mortality [12]. In contrast to conventional bactericidal technology, this novel catalytic approach requires no addition of antimicrobial agents, does not generate secondary pollutants, and is both environmentally sustainable and friendly. It operates efficiently with minimal energy consumption and has a broader range of applications, making it a potential antibacterial technology.

Piezoelectric materials are essential in the piezocatalytic antibacterial process. The rigid texture and toxic lead elements of common piezoelectric materials, such as lead zirconate titanate (PZT), render them challenging to process [13]. Polymer materials, such as polyvinylidene fluoride (PVDF), are biocompatible [14]. However, they are difficult to degrade within the body, which could result in long-term environmental and biosafety concerns. These constraints hinder the utilization of conventional piezoelectric materials in biological systems, prompting the exploration of safer, more sustainable alternative materials. Certain natural materials such as amino acids, proteins, peptides, and wood exhibit intrinsic piezoelectric properties [15,16,17]. These materials are readily accessible, exhibit superior biodegradability and biocompatibility, and can be organically assimilated in the environment without generating secondary contamination. Thus, investigating the uses of piezoelectric biomaterials presents significant potential.

**Figure 1:**
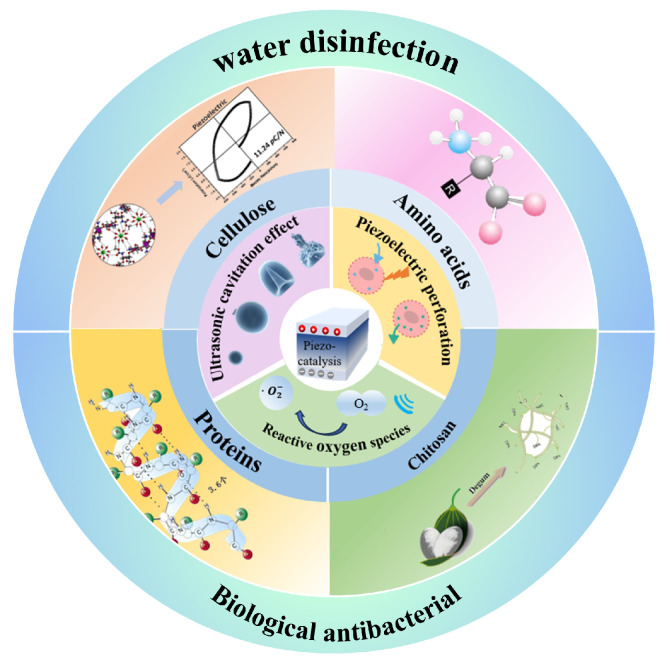
A schematic diagram for the content summary.

This work initially examines the antibacterial mechanisms of piezoelectric catalytic technology and evaluates prevalent piezoelectric biomaterials utilized in antibacterial applications, such as amino acids, cellulose, proteins, and chitosan, as shown in Figure 1. It offers a thorough examination of the practical uses of bio-piezoelectric composites employing piezoelectric catalytic technology to get antibacterial properties. The project is to thoroughly examine mechanically driven catalytic antibacterial studies, consequently promoting innovation and progress in the application of piezoelectric catalytic technology for antibacterial purposes in biomaterials.

## 2. Piezocatalyzed Antibacterial Mechanisms

### 2.1. Ultrasonic-Induced Cavitation Effect

**Figure 2:**
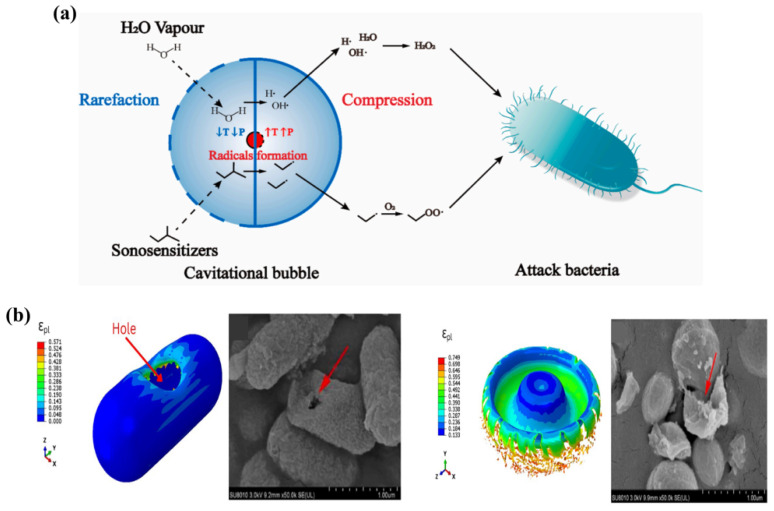
(**a**) Mechanical effect of bubbles on the cell wall of bacteria [20]. (**b**) Finite element analysis and SEM images of *E. coli* cell membrane degradation [26]. Copyright © 2024, 2025, 2020, The Author(s).

The cell membrane functions as both a protective barrier and a central system for essential biological processes [18]. It safeguards the internal structure of the cell and is crucial for material exchange. Disrupting the cell membrane is tantamount to severing the energy and nutrient supply of bacteria [19]. Piezocatalysis can induce cell membrane rupture by exerting substantial mechanical force on the membrane via ultrasound-induced cavitation, as shown in Figure 2a. Ultrasonic waves generate alternating high and low pressure regions as they traverse a liquid. In the low-pressure phase, local pressure may decrease below the saturation vapor pressure of the liquid, resulting in the expansion of existing tiny air nuclei into cavitation bubbles [20]. The collapse of cavitation bubbles generates potent shockwaves and high-velocity micro-jets (up to 110 m/s), creating extreme localized pressure and shear forces that physically compromise bacterial cell integrity [21,22,23]. The frequency of ultrasound plays a crucial role in the degree of bacterial damage by altering cavitation effects. Low-frequency ultrasound is characterized by longer wavelengths and higher energy, allowing bubbles to grow for a longer duration and reach larger sizes [24]. Upon collapse, these bubbles produce more violent reactions, generating stronger localized high temperatures, high pressures, and mechanical impact forces. Therefore, under normal conditions, a lower frequency corresponds to a stronger cavitation effect, resulting in more direct physical destruction of bacteria and a higher lethality rate [25]. Additionally, bacterial morphology influences their response to cavitation under ultrasound, leading to varying degrees of damage. The rod-like morphology of *Escherichia coli* (*E. coli*) renders it highly sensitive to stress concentrations during cavitation impacts, leading to distinct damage modes such as perforation. Conversely, the spherical geometry of *Staphylococcus aureus* (*S. aureus*) distributes pressure more uniformly, necessitating higher-intensity impacts to breach the cell envelope, as depicted in Figure 2b [26]. The spherical morphology of *S. aureus* distributes pressure uniformly throughout its surface, preventing stress concentration, and thus necessitates a “wide-area, high-force” impact to breach the entire overburden. Consequently, to effectively inactivate a particular bacteria, it is essential to determine the ideal amplitude and distribution coefficient of impact pressure based on the mechanical properties and geometry of its cell wall to inflict maximal damage.

### 2.2. Piezoelectric Perforation

**Figure 3:**
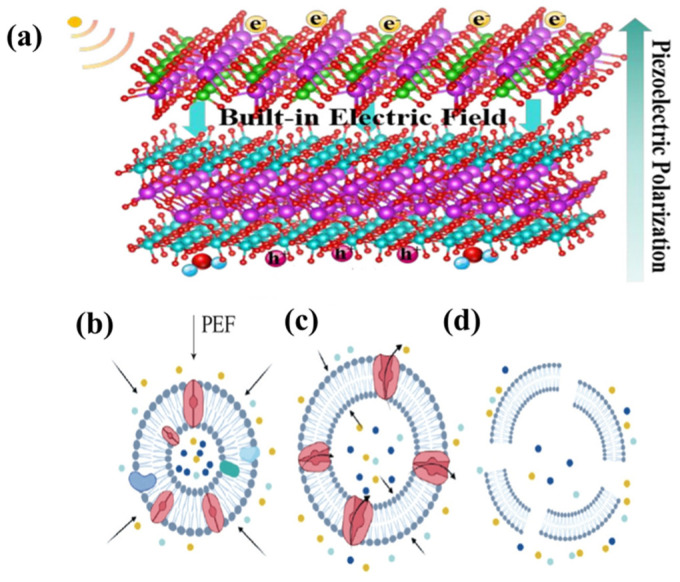
(**a**) Built-in electric field created by piezoelectric polarization. (**b**) Alterations in cell membrane architecture in reaction to an electric field [27]. (**c**) Modified permeability allows water and tiny molecules to enter the cell [27]. (**d**) Cells undergo swelling and membrane rupture, resulting in cell lysis [27]. Copyright © 2025, The Author(s).

Piezoelectric materials possessing non-centrosymmetric crystal structures experience lattice deformation when subjected to external mechanical forces, leading to the relative movement of positive and negative charge centers, hence inducing electrode polarization inside the material [27]. This polarization produces an equivalent quantity of bound charges with opposite signs on the material’s surface and creates a robust local electric field in the surrounding space, as shown in Figure 3a. Exposure of the cell to the electric field causes an extra transmembrane potential across both sides of the cell membrane [28]. The generated potential is overlaid on the cell’s resting membrane potential, collectively determining the total transmembrane voltage at a specific membrane location [29,30]. The electroporation process begins when the membrane lipid bilayer system’s thermodynamic stability is upset due to an absolute value of the local transmembrane potential exceeding a certain threshold. The configuration of phospholipid molecules alters within the electric field. The hydrophilic heads may orient towards the electric field, whilst the hydrophobic tails diverge from one another. This conformational alteration induces localized instability in the phospholipid bilayer, establishing the molecular foundation for electroporation production [31]. Proteins in the cell membrane function as channels for the transfer of substances. In the presence of an electric field, these channels assume an open conformation, enhancing ion mobility across the membrane and modifying its permeability, as shown in Figure 3b. Cations like Na^+^, K^+^, and Ca^2+^ permeate the cell via electrochemical gradients, resulting in a swift elevation in intracellular osmotic pressure. This further diminishes lipid-lipid interactions. The electric field force substantially decreases the energy barrier for hole formation. A little decrease in the energy barrier results in an exponential rise in the likelihood of pore formation, as the rate of pore formation is inversely related to the potential energy barrier [32,33]. This lowering of barriers enables water molecule chains to infiltrate the hydrophobic core of the membrane, thus creating unstable hydrophobic pores, as shown in Figure 3c,d. Excessive quantity or size of holes in the cell membrane results in irreversible membrane damage. This results in the efflux of intracellular components, ultimately leading to cellular demise.

### 2.3. Bacterial Inactivation Mediated by ROS

**Figure 4:**
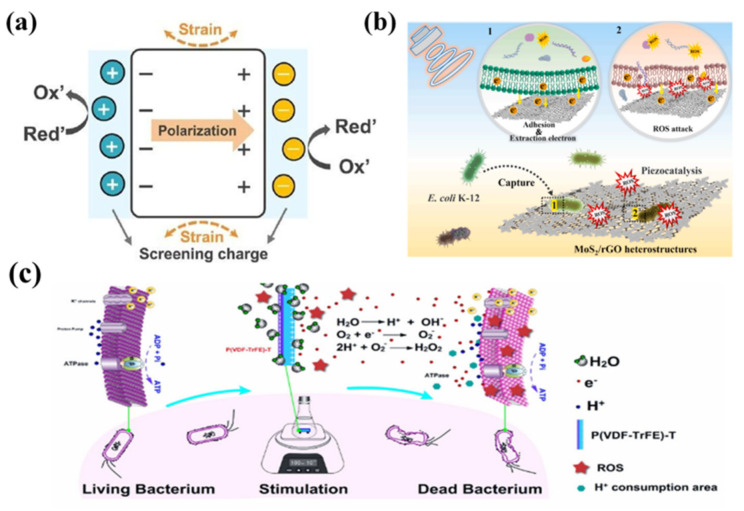
(**a**) The generation of release screening charges to produce ROS when the material experiences compressive strain [37]. (**b**) Mechanism of piezocatalytic inactivation of *E. coli* K-12 by MoS_2_/rGO [38]. (**c**) Antibacterial mechanism of self-powered flexible nano-piezoelectric film [40]. Copyright ©2022, 2025, The Author(s).

In the aqueous phase system, hydroxyl radical (·OH), superoxide radical (·O_2_^−^), and single linear oxygen (^1^O_2_) are three ROS with strong oxidizing properties [34]. Piezocatalysis, as a new catalytic technology, is able to collect mechanical energy from the environment and achieve efficient sterilization by generating ROS [35]. The basic idea is that a material with a piezoelectric effect has a non-centrosymmetric deformation in its internal crystal structure when stress is applied. This causes the positive and negative charge centers to separate, producing h^+^ and e^−^ [36]. These carriers are driven by an internal piezoelectric field to migrate to the material surface and participate in interfacial redox reactions. The holes can oxidize adsorbed H_2_O or OH^−^ to generate ·OH, as shown in Figure 4a [37]. Meanwhile, the electrons can reduce adsorbed O_2_ to generate ·O_2_^−^. This strong oxidizing ROS can destroy the bacterial cellular structure, thus realizing antimicrobial efficacy:

(1) $${OH}^{-}+h^{+}\to \cdot OH$$

(2) $$O_{2}+e^{-}\to \cdot O_{2}^{-}$$

(3) $$bacteria\;ROS(\cdot O_{2}^{-}/\cdot OH)\to dead\;bacteria$$

Activated oxygen induces rapid sterilization via a “multi-target-multi-step” cascade, as shown in Figure 4b [38]. Initial oxidation of membrane phospholipid polyunsaturated fatty acids instigates lipid peroxidation, resulting in membrane perforation and a sudden increase in permeability; this is followed by significant leakage of K^+^, Mg^2+^, and ATP, leading to ATP depletion and osmotic pressure imbalance. ATP depletion and osmotic pressure imbalance together exacerbate the damage, resulting in the bacteria’s immediate loss of reparative capacity and subsequent death. At the same time, ROS will also oxidative damage proteins and enzymes on the cell membrane, inhibiting their activities, interfering with the normal cellular metabolism and respiration process, and blocking the electron transport chain, further exacerbating the generation of ROS and oxidative stress [39]. In addition, ROS are able to penetrate the cell membrane and enter the cell interior, combining with DNA and causing DNA strand breaks and base modifications, destroying the integrity of the genetic information and the process of replication and transcription, and hindering cell division and proliferation. ln the cell wall, ROS can inhibit the activity of periplasmic enzymes, weaken the synthesis and repair ability of the cell wall, and make the cell wall structure become fragile; ultimately, under the synergistic damage of multiple targets, such as the cell membrane, proteins, DNA, and the cell wall, the normal physiological functions of bacterial cells are severely damaged, and they are unable to maintain their life activities, which leads to the death of the bacteria, as illustrated in Figure 4c [40]. For example, Yang et al. prepared a self-powered flexible nano-piezoelectric membrane, using electrostatic spinning method to prepare this poly(vinylidene fluoride-trifluoroethylene) (PVDF-TrFE) piezoelectric membrane, which generates an electric field under mechanical stimulation and breaks the hydrogen bond in H_2_O, thus generating ROS, which can lead to the destruction of the cell membrane by increasing intracellular oxidative stress and cell membrane permeability to kill bacteria [41].

Ultrasonic frequency, as a key parameter in the piezocatalysis process, can be rationally adjusted to modify the piezocatalytic performance. At a lower ultrasonic frequency, bubbles undergo a longer growth period, and the larger bubbles lead to a greater degree of compression on the piezoelectric material upon collapse, which in turn triggers more polarized charges and active charges to interact with the adsorbed species on the surface of the piezoelectric body. When the frequency is too low, the poor periodicity of the lag in the collapse force will shield the piezoelectric field, hindering the occurrence of the piezoelectric catalytic reaction [42]. Therefore, it is crucial to select an appropriate ultrasonic frequency in the piezocatalytic process. The piezocatalytic performance is optimal when the ultrasonic frequency is very close to the natural frequency of the piezocatalyst. This is because the shock wave generated by the collapse of cavitation bubbles superimposes in phase with the large-amplitude vibration of the material itself caused by resonance, which maximizes the instantaneous mechanical stress acting on the material and further amplifies the piezoelectric effect.

## 3. Prevalent Biological Substances Exhibiting Piezoelectric Characteristics

### 3.1. Amino Acids

**Figure 5:**
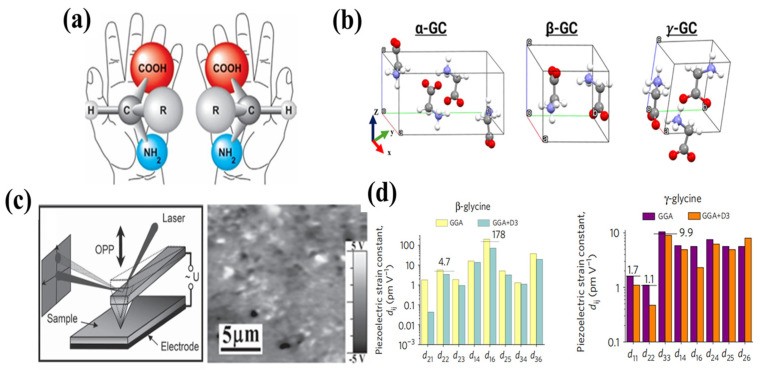
(**a**) Molecular structure of amino acids. (**b**) Structures of Glycine in different crystalline forms. (**c**)Piezoelectric response curve of γ-Glycine [50]. (**d**) Comparison chart of piezoelectric coefficients for γ-Glycine and β-Glycine [52]. Copyright ©2017, 2025, 2026, The Author(s).

Amino acids are the essential constituents of biological macromolecules like proteins and display structure-dependent piezoelectric characteristics [43]. The molecular formula of amino acids is represented as R-CH(NH_2_)-COOH, comprising a side chain group, an amino group, a hydrogen atom, and a carboxyl group [44]. Amino acids can generate three-dimensional crystalline formations. An amino acid exhibits piezoelectricity when its crystal structure corresponds to one of the 20 piezoelectric point groups [45]. Piezoelectricity is related to two basic structural properties of amino acids: chirality is the first. With the exception of glycine (R=H), the α-carbon (C*) of amino acids functions as a chiral center, linking four unique groups. This produces chiral symmetry groups with left-handed (L) and right-handed (D) alterations (Figure 5a). Chiral molecules cannot be fully superimposed on their mirror copies by rotation or translation in three-dimensional space, thus violating the structure’s core symmetry [46]. Non-centrosymmetry is essential for crystals to demonstrate piezoelectricity. Consequently, chirality establishes the structural foundation for amino acid crystals to produce piezoelectric reactions. Lemanov et al. conducted a comprehensive analysis of amino acids in 20 proteins using nuclear quadrupole resonance spectroscopy, revealing that 16 of these amino acids exhibit measurable piezoelectric activity. L-methionine has a piezoelectric response at temperatures below 210 K [47]. Furthermore, atoms with significant electronegativity differences in amino acid molecules (e.g., N, O, H) and charge separation in zwitterionic forms (NH_3_^+^ and COO^−^) result in pronounced intrinsic dipole moments inside the molecules [48]. Upon exposure to mechanical stress, the dipole moment undergoes directional changes, leading to electrical polarization. This delineates the physical foundation for the piezoelectric response demonstrated by amino acids at the molecular level. Glycine is the most elementary amino acid in the sequence, displaying three unique crystalline forms: α, β, and γ phases [49]. The α-glycine crystal structure comprises dipolar zwitterionic glycine molecules organized into centrally symmetric cyclic dimers, classified within a centrally symmetric point group and devoid of piezoelectric properties. In contrast, β-glycine exhibits net polarization along its longitudinal axis, while γ-glycine polarizes spontaneously along its perpendicular axis. Both demonstrate non-centrally symmetric point groups and exhibit piezoelectric properties [48]. Alejandro et al. utilized conductive probes to provide an alternating voltage to the sample surface, thereby producing subtle deformations via the inverse piezoelectric effect. Figure 5b,c illustrates that the γ-glycine area had a pronounced, heterogeneous piezoelectric response contrast. The quantification of γ-glycine’s piezoelectricity by PFM resulted in an effective piezoelectric coefficient of around 10 pm V^−1^ [50]. Recent studies demonstrate that β-glycine possesses remarkably robust piezoelectric characteristics. Guerin et al. examined the piezoelectric responses of β- and γ-glycine by DFT simulations. Notably, β-glycine (space group P_21_) exhibits a monoclinic angle of 112° (the highest among all amino acids), leading to exceptionally low stiffness along a particular shear plane (aligned with the c_66_ direction; c_66_ = 1.3 GPa). During shear deformation along this “soft” direction, molecular dipoles can effectively reorganize collectively, producing significant piezoelectric polarization. The piezoelectric coefficient of β-glycine was measured using a quasi-static d_33_ device, yielding a value of d_16_ = 178 pm V^−1^. Despite γ-glycine (space group P3_2_) having a greater net dipole moment, its molecular arrangement contributes to increased stiffness, resulting in a reduced piezoelectric coefficient of 9.9 pm V^−1^ [51]. The piezoelectric coefficient of β-glycine is roughly tenfold greater than that of γ-glycine. The inherent piezoelectric activity of amino acids has surfaced as a viable piezoelectric material for biodegradable and biocompatible applications; nevertheless, their comparatively low piezoelectric coefficients constrain practical use. Wu et al. proposed a fluorination approach to address this constraint and improve the piezoelectric characteristics of amino acid crystals. Hydrogen atoms on the aromatic rings of L-tryptophan, L-phenylalanine, and N-Cbz-L-phenylalanine were replaced with fluorine, resulting in enhanced piezoelectric coefficients. Density functional theory calculations reveal that fluorination promotes polarization by modifying the molecule dipole moment. As a result, these fluorinated crystals demonstrate piezoelectric coefficients of 50.36 pm/V, exceeding those of other organic piezoelectric materials such PVDF, poly(L-lactic acid) (PLLA), and gelatin. This study proposes a flexible method for improving the piezoelectric characteristics of biomaterials, hence expanding their range of applications [52].

### 3.2. Cellulose

**Figure 6:**
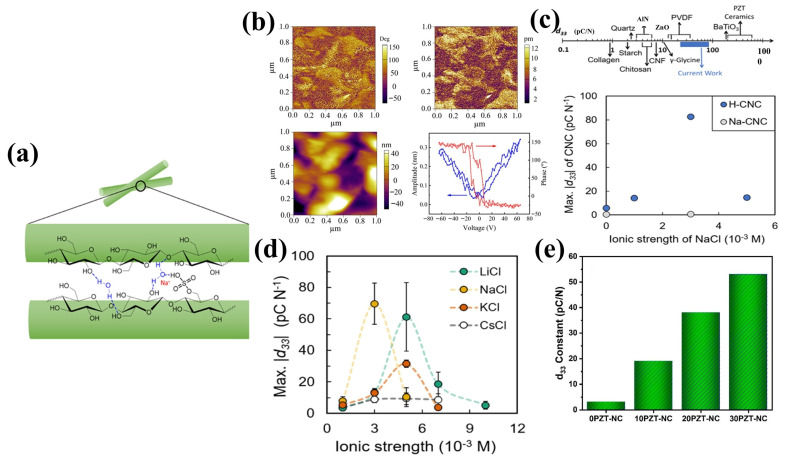
(**a**) Molecular configuration of cellulose polymers. (**b**) Phase image of cellulose suspension [53]. (**c**) Maximum value from at least three replicate measurements taken from different areas of the same film |d_33_| value [55]. (**d**) The maximum values of |d_33_| in CNC films cast from suspensions with varying ionic strengths of NaCl [58]. (**e**) Piezoelectric constants of pure NC and PZT-NC films [60].

Cellulose is a macromolecular polysaccharide composed of glucose units, insoluble in water and most organic solvents, serving as the primary component of plant cell walls (Figure 6a). Cellulose, the most abundant biomaterial in nature with extensive resources, possesses unique physical and chemical properties that make it an excellent candidate for piezoelectric materials. Cellulose generally manifests in four crystalline forms: cellulose I, II, III, and IV [53]. Cellulose I is the most abundant type in nature, often found in two crystalline configurations: the triclinic Iα and the monoclinic Iβ. The space groups for these two types are P_1_ and P_21_, respectively. P_1_ and P_21_ are classified inside the chiral space group category among the 230 space groups [54,55]. Chiral space groups lack symmetry centers. The non-centrosymmetric structural properties of these two forms dictate their capacity to produce piezoelectric effects when subjected to mechanical stress. The piezoelectric properties of cellulose were initially confirmed by positive and reverse piezoelectric testing systems, establishing piezoelectric constants of 10^−13^N/C for species such as Pinus massoniana [56]. This phenomenon arises from the systematic organization of cellulose microfibrils within cell walls. The crystalline domains of cellulose display a monoclinic crystal structure characterized by point group symmetry C2. The lack of inversion symmetry in the crystalline domains results in an intrinsic electric dipole moment. When the material deforms under stress, alterations in the dipole moment are observed macroscopically as electric polarization, known as the piezoelectric response [57]. Additionally, cellulose chains consist of glucose units interconnected by β-1,4-glycosidic linkages, with several hydroxyl groups along the chain that can establish intra- and inter-chain hydrogen connections. The hydrogen bonds and hydrophobic interactions compel cellulose chains to align in a parallel orientation, leading to a macroscopic arrangement where all reducing ends are oriented on one side and non-reducing ends on the other side, therefore enhancing the net dipole moment. This highly organized, non-centrosymmetric chain arrangement allows cellulose to produce substantial piezoelectric signals, even at the nanoscale (e.g., CNC, CNF). Yang et al. utilized piezoelectric force microscopy (PFM) to assess the intrinsic piezoelectric characteristics of nano-cellulose. The distinctive amplitude butterfly loops and 180 Ω phase hysteresis behavior confirmed the piezoelectric properties of nano-cellulose, resulting in a quantifiable piezoelectric constant (d_33_ = 4.4 pm/V), as shown in Figure 6b [58]. The diminished piezoelectric coefficient of cellulose is a considerable obstacle. Surface chemical alteration can significantly augment its piezoelectric effect. Miao et al. transformed sulfate-hydrolyzed cellulose from the sodium form (Na-CNC) to the acid form (H-CNC), protonating surface sulfate groups (-OSO_3_H) to enhance interparticle hydrogen bonding interactions. Hydrogen bonds, as polarity-sensitive structural units, easily deform and polarize under external stresses, acting as a primary source of piezoelectric response. Enhancing the ionic strength in the suspension with the addition of low-concentration salts (e.g., 3 mM NaCl) optimizes the electrostatic conditions at the cellulose interface. This enhances polarization efficiency and charge transfer across the hydrogen bond network, increasing the piezoelectric coefficient (d_33_) from under 1 pC/N to a peak of roughly 82.6 pC/N. Following several cycles, the coefficient stabilizes at around 29 pC/N, comparable to commercial PVDF, as shown in Figure 6c,d [59]. Moreover, the fabrication of cellulose-based piezoelectric composites can augment the piezoelectric characteristics of cellulose. Latif et al. accomplished highly aligned nano-cellulose fiber alignment by extrusion-based 3D printing. Through the application of magnetically induced polarization, they aligned PZT piezoelectric domains in designated orientations, resulting in a composite system characterized by both highly directed structures and uniformly distributed piezoelectric phases. This technique improves the effectiveness of force-to-electricity conversion by facilitating effective stress transfer at the PZT-cellulose contact, as per the Furukawa model. The synthesized nano-cellulose film with 30 wt% PZT demonstrated a significant electric constant (d_33_ = 53 pC/N), surpassing pure cellulose by 1.8 times and commercial PVDF by 2.3 times, as shown in Figure 6e [60].

### 3.3. Proteins

**Figure 7:**
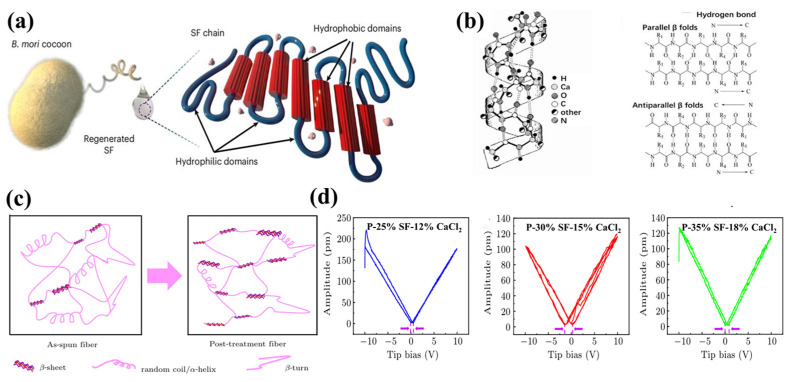
(**a**) Structural characteristics of silk proteins [63]. (**b**) Molecular structure of keratin: α- helix and β-fold. (**c**) The schematics of conformation transformation of SF molecules during the post-treatment process [65]. (**d**) Butterfly curves of different SF fibers [70]. Copyright ©2026, 2026, 2023, The Author(s).

Proteins are natural biological polymers formed from more than 20 amino acids connected by peptide bonds in diverse ratios, functioning as vital constituents of all human cells and tissues [61]. Amino acids, the essential constituents of proteins, are predominantly chiral compounds. This chirality occurs due to the α-carbon atom forming bonds with four unique groups, leading to a molecular structure devoid of central symmetry [62]. The linkage of these asymmetric amino acids via peptide bonds results in the formation of a polar structure inside a polypeptide chain. Furthermore, the peptide bond (-CO-NH-) exhibits a dipole moment, with the orientation of the dipole orientated approximately from hydrogen to oxygen α-helix and β-sheet are the two principal secondary structures of proteins (Figure 7a,b) [63]. In these secondary structures, hydrogen bonds are organized in a highly ordered and unidirectional fashion, creating a significant macroscopic dipole moment [64]. External forces exerted on proteins induce the stretching, bending, or reconfiguration of hydrogen bonds, therefore modifying the strength and direction of these dipoles. This produces net electrical polarization, facilitating the transformation of mechanical energy into electrical energy. This dynamic network created by weak connections is essential for proteins to demonstrate piezoelectricity. Among the various categories of proteins, prevalent piezoelectric proteins like collagen, keratin, and fibrinogen have been documented to exhibit piezoelectric capabilities. Keratin is a member of the fibrous structural protein family. The monomers assemble into bundle structures through intermediate filaments, with peptide chains assuming either α-helix or β-sheet conformations. The piezoelectricity of keratin principally derives from the inherent dipoles of α-helices and their configuration. In α-keratin, two right-handed α-helices coalesce to create a more stable left-handed superhelical configuration. Throughout this winding process, the axes of the two α-helices remain parallel and co-directional [65]. This indicates that the positive poles (N-termini) of both α-helix dipoles orient toward one end of the fiber, whilst their negative poles orient toward the opposite end. Their dipole moments thus align in the same direction instead of negating one another. When keratin fibers experience axial tension or compression, external forces modify the pitch, diameter, and spatial arrangement of the α-helices. This deformation directly causes slight alterations in the density and orientation of peptide bond dipole moments within the α-helices, thereby affecting the aggregate of all aligned macroscopic dipole moments in the material [66]. Gauss’s law states that this change in polarization intensity generates a net charge on the surface of the substance. Zhao et al. utilized electrospinning to produce Bi_4_LaTi_3_FeO_15_ (BLTF) nanofibers (NFs) as a wool keratin-derived biocompatible piezoelectric nanogenerator. The BLTF NFs nanogenerator generates an output voltage of 0.14 V and an output current of 41 nA, with the produced power exhibiting remarkable reproducibility [67]. The piezoelectric properties of sericin fundamentally contrast with the “dipole orientation” mechanism of keratin, principally depending on ionic polarization and the deformation of hydrogen bond networks. In sericin, sequences abundant in glycine (Gly), alanine (Ala), and serine (Ser) autonomously organize into antiparallel β-lamellae [68]. The sheets are laterally reinforced by robust intermolecular hydrogen bonds, resulting in stiff, almost inextensible nanocrystals. In the β-sheets, polar atoms (C=O, N-H) of the peptide backbone engage in the hydrogen bond network, with their charges comparatively “fixed.” Under external stress, the rigid β-sheets maintain relative stability; nonetheless, this results in slight relative slippage or displacement between the sheets. This displacement modifies the distribution of charged ions or polar groups inside the interlayer regions, producing piezoelectric voltages at the material’s extremities. Increased β-sheet content results in enhanced material rigidity, and sufficient stiffness is essential for the effective transmission of mechanical stress (Figure 7c). Consequently, the β-sheet composition dictates the intensity of piezoelectricity. Zhang et al. synthesized pure silk fibroin piezoelectric fibers with dry spinning technology, employing a spinning solution system composed of pure silk fibroin, CaCl_2_, and formic acid (FA). Through the modulation of calcium ion and silk fibroin (SF) concentrations, alongside stretching and ethanol treatment, they augmented the β-sheet content in SF fibers by regulating the condensed state structure of SF. Post-treatment, the SF fibers demonstrated a maximum piezoelectric coefficient d_33_ of 3.24 pm/V and a peak output voltage of 27 V, indicating a substantial enhancement in performance compared to the initial SF fibers [69]. In collagen, polypeptide chains organize into a coiled triple helix configuration, resulting in rod-like structures. The aligned structure of collagen promotes piezoelectric effects. ESM collagen piezoelectric nanofibers synthesized by et al. demonstrated a peak dielectric constant at a PEO:SEP ratio of 1:1, surpassing the majority of documented biomolecular membranes, as shown in Figure 7d. The elevated dielectric constant is ascribed to hydrogen bonding with PEO, which promotes the polarized alignment of collagen along PEO polymer chains during electrospinning (or electro polarization) under electrical tension. The engineered sensors demonstrated unique piezoelectric response outputs under different pressures and frequencies, validating their superior capacity to detect dynamic frequencies and pressures [70].

### 3.4. Chitosan

**Figure 8:**
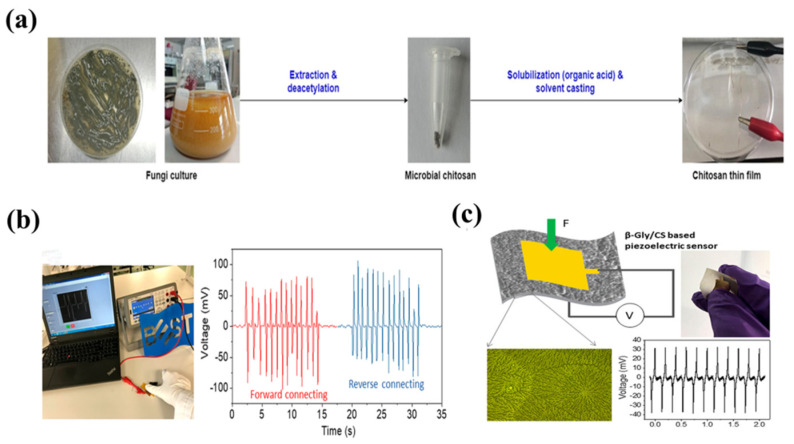
(**a**) Biological synthesis of microbial chitosan using fungal biomass [72]. (**b**) Experimental arrangement for evaluation of the β-Gly/CS-based pressure sensor. (**c**) Flexible sensors based on Gly/CS films [75]. Copyright ©2021, 2017, The Author(s).

Chitosan is a natural biopolymer produced from the deacetylation of chitin, consisting of N-ethylglucosamine monomers connected by β-1,4-glycosidic linkages, forming a naturally occurring fundamental polysaccharide [71]. Chitosan presents two crystalline variants: α-chitosan and β-chitosan. α-chitosan exhibits an uneven helical configuration, whereas β-chitosan displays a highly crystalline fiber structure [72]. The piezoelectric action of chitosan principally derives from its molecular architecture. Upon exposure to external pressures, hydrogen bonds and chemical bonds within its molecules experience minor deformation, resulting in a modification in the internal charge distribution and the production of electrical signals [73]. E. Praveen et al. observed that the fundamental unit of chitosan is D-glucosamine, a chiral molecule. This chemical characteristic automatically prohibits the polymer chains derived from it from having a center of symmetry. Based on XRD studies and a review of the literature, the scientists deduced that chitosan is likely classified within the orthorhombic crystal system, namely with the space group P_21_. This space group is classified as a non-centrosymmetric point group, hence fulfilling the crystallographic criteria necessary for the generation of piezoelectricity. They additionally quantified the d_33_ values of formate chitosan at varying pressures and temperatures. The findings demonstrate that at 330 K and a 5-ton load, the d_33_ value attained 18.4 pC N^−1^ [74]. Ahmad et al. isolated chitosan from the fungal biomass of aspergillus. Structural research indicated that chitosan exhibits a non-centrosymmetric crystal structure, suggesting its potential as a sustainable source for piezoelectric materials. Moreover, chitosan derived from fungal cell walls is easily scalable for commercial manufacturing, providing an eco-friendly industrial approach for piezoelectric applications (Figure 8a). Furthermore, Amran et al. synthesized a chitosan membrane from chitosan produced from A. niger and examined its piezoelectric capabilities. The findings demonstrated that the piezoelectric coefficient d33 of the microbial chitosan membrane attained 10 pC N^−1^, comparable to that of conventional PVDF membranes [75]. Scientists have concentrated on augmenting the piezoelectric characteristics of chitosan due to its intrinsically low piezoelectric response. Amit Nain from the Indian Institute of Science synthesized chitosan (CHT) films using solvent casting and subsequently crosslinked them in an alkaline solution. Sodium hydroxide facilitated deprotonation, resulting in enhanced intramolecular hydrogen bonding and improved mechanical characteristics. With applied stresses ranging from 1 to 16 N, the output voltage progressively ascended from 0.9 V to 1.8 V. The CHT film demonstrated considerable antibacterial and anti-inflammatory effects when subjected to ultrasonic stimulation, along with the suppression of inflammatory cytokines, as shown in Figure 8b,c. Furthermore, chitosan is frequently integrated with other piezoelectric substances to make hydrogels. Li et al. integrated piezoelectric poly(dopamine)-modified barium titanate (PDA-BaTiO_3_,PBT) nanoparticles and hydroxyapatite (PHA) nanoparticles into chitosan/gelatin hydrogels to develop an innovative piezoelectric hydrogel scaffold for bone tissue engineering. Experimental findings validated that this piezoelectric hydrogel scaffold exhibits self-power generation, enhances endogenous growth factor secretion, displays immunomodulatory properties, and demonstrates angiogenic and osteogenic potential.

## 4. Fabrication Strategies for Piezoelectric Biocomposites

SF piezoelectric materials predominantly exist as films, fibers, and composite hydrogels. Various preparation techniques during the manufacture of SF piezoelectric materials influence the material’s piezoelectric capabilities and mechanical qualities differently. During the production and spinning of SF films, physical shearing and stretching induce a certain degree of stretching orientation in the SF. This enables the elongated SF molecule chains to align preferentially in particular orientations, resulting in an anisotropic microstructure. This systematic configuration markedly improves the material’s mechanical strength and toughness while simultaneously optimizing its piezoelectric characteristics; the strongly orientated β-folded crystal domains may respond more efficiently to external stress, producing more robust piezoelectric signals.

### 4.1. Spin Coating

**Figure 9:**
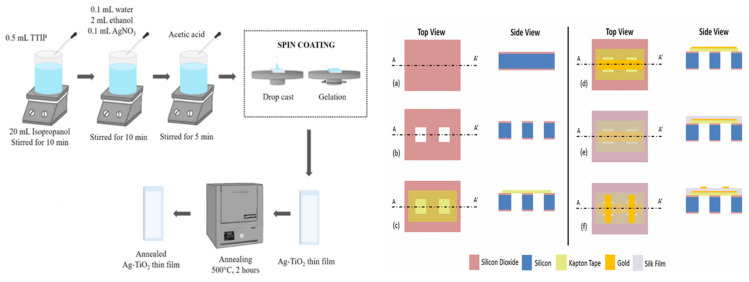
Schematic diagram of the process flow for preparing pressure sensors using the spin-coating method.

Spin coating is a method that use centrifugal force to create homogeneous layers on substrate surfaces. Spin coating, as an efficient and cost-effective film preparation technique, has exhibited extensive application potential across various domains in recent years [76]. The procedure commences with the fast dispersion of the solution over the entire substrate surface using centrifugal force. Consequently, the solvent commences to evaporate swiftly. As solvent evaporation advances, the viscosity of the solution consistently escalates, resulting in increased viscous forces and a gradual reduction in flow rate. Ultimately, these forces attain a dynamic equilibrium in which the film thickness remains relatively constant throughout time. Subsequently, the film is subjected to a higher-temperature environment, where residual solvent persists in evaporating, resulting in a significant increase in solution concentration. Solute molecules or particles progressively converge and coalesce, ultimately forming a solid-state film by processes such as gelation, vitrification, or crystallization [77]. For instance, Yuan et al. applied a sericin precursor solution onto silicon wafers using spin coating at 1000 rpm for 30 s, as depicted in Figure 9. The films were further baked in a vacuum oven at temperatures between 50 °C and 90 °C for durations of 15 min to 2 h, resulting in ultra-smooth sericin films with a thickness of 900 nm ± 25 nm. PFM studies of the film’s piezoelectric response indicated an average d33 value of around 56.7 pm/V for the SF piezoelectric film. During spin coating, significant inertial forces generate shear and tensile stresses in the fluid, elongating the SF film and improving the alignment of β-folds within it. CNF was concentrated to 7 wt% in an oven at 55 °C and agitated at room temperature for 24 h. The solution was evenly distributed on a PTFE plate and allowed to rest in an 80 °C oven for 2 h, resulting in a cellulose piezoelectric film [78].

### 4.2. Electrochemical Deposition

Electrochemical deposition is a liquid-phase technique that employs electrochemical processes to produce multifunctional films on material surfaces. The fundamental principle entails the movement of charged ions (or particles) in solution towards the electrode surface when subjected to an electric field. Through reduction (electron gain) or oxidation (electron loss) events, these ions convert into solid substances and deposit as thin sheets [79]. Vanjari et al. utilized electrodeposition to create evenly deposited, ultra-smooth SF films. During deposition, SF molecules experience electric field pressures that augment their orientation and promote β-sheet folding. Subsequent to deposition, the sample was submerged in an 80 vol% methanol-water solution for 5 min to augment crystallinity, hence enhancing the piezoelectric sensitivity of the SF film, yielding a d_33_ value of 8.39 pm/V.

### 4.3. Self-Assembly Strategy

**Figure 10:**
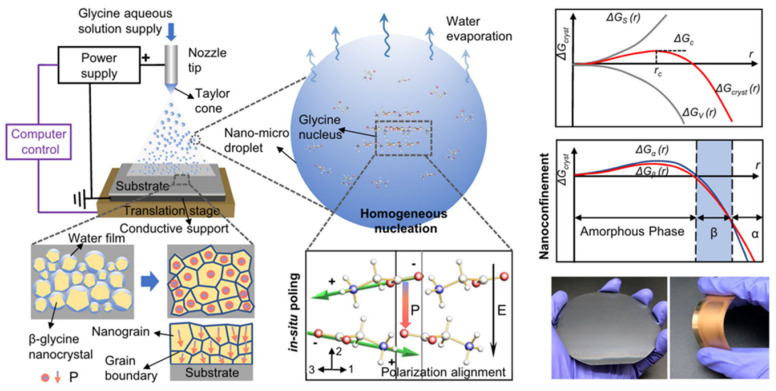
Mechanism of Self-Assembly Strategy for Preparing Piezoelectric β-Glycine Nanocrystalline Thin Films [81].

Self-assembly techniques are a method for producing films with superior high-voltage electrical performance. This technique actively employs two essential external control parameters to dictate the final film structure, rather than passively awaiting molecule assembly. The nanoconfinement effect is utilized to regulate crystal phase and initial orientation. In the initial crystallization phase, the least stable crystal form emerges preferentially owing to its diminutive size and prevailing surface energy [80]. In the limited volume of nanodroplets, the critical nucleation free energy of β-glycine is inferior to that of the α phase, as illustrated in Figure 10. resulting in the favored emergence of metastable yet piezoelectric β-phase crystals [81]. In the absence of this size constraint, traditional evaporation produces solely the α phase. Structural orientation alone is inadequate; the internal dipole moments (positive and negative charge centers resulting from uneven charge distribution in molecule chains) must align evenly in a single direction to exhibit macroscopic piezoelectricity. Nanoconfinement facilitates film production, such as by solvent evaporation or gel curing, while a robust direct current electric field is concurrently supplied for in situ polarization. The electric field force influences dipoles in molecule chains or crystals, allowing them to surpass thermal motion and intermolecular forces. This compels the dipoles to spin and orient themselves along the direction of the electric field, usually corresponding to the thickness direction of the film. The electric field force influences dipoles in molecule chains or crystals, allowing them to surpass thermal motion and intermolecular forces. This leads to the internal dipole moments being consistently aligned with the direction of the electric field. The two characteristics immediately cause the film to display a piezoelectric voltage constant (d_33_) markedly superior to that of disordered films or those subjected to singular treatment methods in the thickness direction. The piezoelectric strain coefficient d_33_ of β-glycine attains 11.2 pm V^−1^, but the piezoelectric voltage coefficient g_33_ is as elevated as 252 × 10^−3^ Vm N^−1^, far surpassing the majority of biological piezoelectric materials [82].

### 4.4. Electrospinning

**Figure 11:**
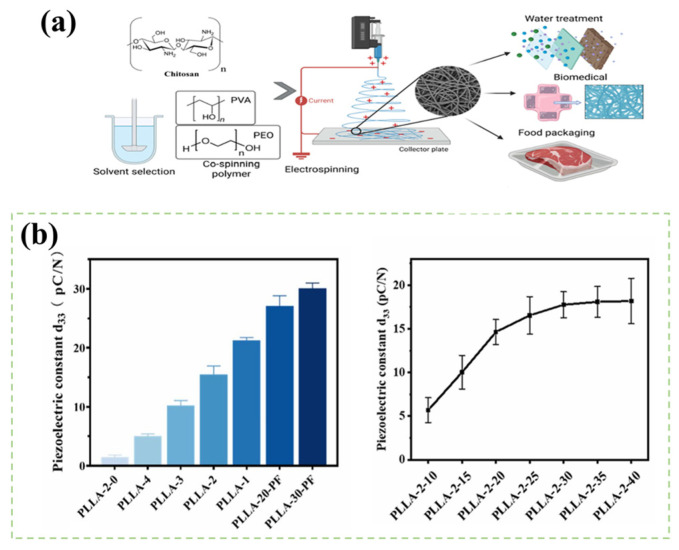
(**a**) The process of preparing biofibers via electrospinning. (**b**) Comparison of piezoelectric coefficients for materials obtained at different spinning solution concentrations.

Electrospinning is a premier technique for fabricating piezoelectric nanofibers, as it simultaneously provides the high electric field required for dipole alignment and the mechanical stretching needed for phase transformation (e.g., α to β phase) [83]. During spinning, the jet transports high-density charges, producing a robust intrinsic electric field while being elongated by electrostatic repulsion at an exceedingly high strain rate (~10^4^ s^−1^). The high flow-field stretching predominates the crystalline phase transformation, compelling PVDF molecular chains to shift from the randomly coiled or helical (TGTG’) conformation of the α phase to the completely stretched, fully trans (TTTT) β crystalline phase conformation, as illustrated in Figure 11a [84]. The enduring strong electric field collaborates with the stretching process. It not only stabilizes the β-phase conformation but also vitally directs the inherent C-F dipole moments within the nascent β-phase crystals to align with the electric field direction (usually parallel to the fiber axis), facilitating in situ polarization [85]. Consequently, electrospinning combines material shape, piezoelectric phase induction, and initial electric domain orientation in one process, producing nanofiber membranes with elevated β-phase content and superior piezoelectric response. Li et al. electrospun a composite solution of PLLA and glycine, employing high voltage to create a glycine shell layer on the fiber surface, with PLLA molecules constituting the fiber core. The creation of this core–shell structure arises from hydrogen bonding between the hydroxyl (-OH) groups in glycine molecules and the carbonyl (C=O) groups in PLLA molecules. This interaction not only directs the systematic assembly of PLLA molecule chains but also facilitates β-phase formation. Liu et al. conducted a detailed examination of the influence of spinning solution composition, electric field intensity, feed and receive velocities, and ambient conditions on the piezoelectric characteristics of fibers. The piezoelectric constants obtained were 28.5, 24.3, 19.9, and 19.7 pC/N, respectively (Figure 11b), demonstrating that a reduction in solution concentration markedly improves the piezoelectric performance of PLLA nanofiber membranes. Modulating the volume of the PLLA-2 spinning solution influenced the thickness of the nanofiber membrane, thus affecting its piezoelectric coefficient. The d_33_ value of the PLLA nanofiber membrane exhibited a linear increase with thickness. Nonetheless, as the volume of the PLLA-2 spinning solution increased further, the rate of d_33_ value enhancement diminished. The d_33_ value of PLLA-2-40 attained a peak of 18.7 pC/N [86]. In addition, the influence of the conductivity of the spinning solution on the piezoelectric constant was examined. An increase in conductivity correlates with an increase in the piezoelectric constant. This enhancement is attributed to the increased electrical conductivity, which leads to a reduction in fiber diameter. The finer fibers exhibit stronger polarity, thereby improving their sensitivity to external forces and endowing them with a higher piezoelectric response. As a result, mechanical vibration induces the fibers to produce an increased quantity of piezoelectric charge and display an elevated piezoelectric constant.

### 4.5. Physical-Chemical Crosslinking Method

**Figure 12:**
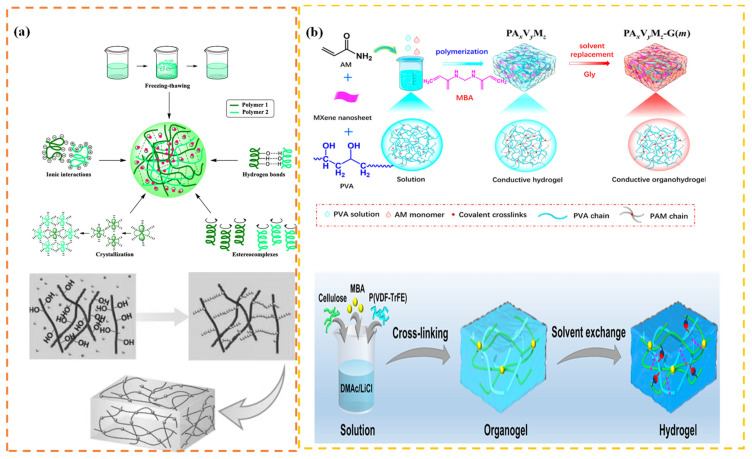
Procedure for synthesizing hydrogels via (**a**) physical and (**b**) chemical crosslinking techniques [88,89]. Copyright ©2025, 2023, The Author(s).

Physical crosslinking is a prevalent technique for synthesizing hydrogels. It establishes a three-dimensional network structure by intermolecular forces, in contrast to the covalent bond connections of chemical crosslinking [87]. The fundamental principle of physical crosslinking is in the synergistic effects of many non-covalent bonds, predominantly encompassing hydrogen bonding, electrostatic/ionic interactions, crystalline domain crosslinking, host-guest recognition, and supramolecular assembly. Hydrogen bonding is a predominant interaction in physical crosslinking, largely characterized by the establishment of hydrogen bonds between hydrogen-donating groups (e.g., -OH, -COOH, -NH_2_) and hydrogen-accepting groups (e.g., -O-, -C=O, -N-) on polymer chains, as illustrated in Figure 12 [88]. When the concentration of polymer chains is sufficiently elevated and conditions (e.g., lowered temperature, pH fluctuations) are conducive, hydrogen donors and acceptors on the chains start to identify and converge, resulting in the formation of singular or localized hydrogen bonds. These bonds briefly tether specific segments of two or more strands together. A self-polarized double-network cellulose/P(VDF-TrFE) (DNCP) hydrogel was reported, wherein flexible cellulose chains are integrated with stiff piezoelectric polymer (VDF-TrFE) chains via covalent bonding, hydrogen bonding, and dipole interactions. The intrinsic polarity of cellulose molecules fosters robust dipole interactions with P(VDF-TrFE), facilitating the nucleation of P(VDF-TrFE) into the electroactive β phase. This enables a β phase content of 75.1% in P(VDF-TrFE) without necessitating further polarization. This DNCP hydrogel sensor demonstrates exceptional self-powered sensing capability [89]. Ionic crosslinking constitutes a notable type of physical crosslinking, generally accomplished by electrostatic interactions between multivalent metal ions and anionic polymers. For example, when sodium alginate, a natural polysaccharide abundant in carboxylate groups, interacts with calcium ions (Ca^2+^), it creates a “egg-crate” configuration with the G-units (guluronic acid) of sodium alginate, swiftly crosslinking into a gel.

Chemical crosslinking establishes permanent covalent linkages between polymer chains, resulting in durable three-dimensional network architectures. This technique generally necessitates the incorporation of chemical crosslinking agents or the attainment of crosslinking through certain processes. In contrast to physical crosslinking, the covalent bond network established through chemical crosslinking is enduring and irreversible. As a result, chemically crosslinked hydrogels often demonstrate enhanced mechanical strength, stability, and swelling resistance [90]. Free radical polymerization is a widely employed chemical crosslinking technique that generally entails the production of reactive radicals by initiators to initiate chain polymerization reactions of monomers with double bonds [91]. Dumitrescu et al. manufactured a hydroxyapatite-potassium-sodium niobate-chitosan (HA-KNN-CSL) biocomposite by incorporating hydroxyapatite nanopowder (HA) and submicron potassium-sodium niobate powder (KNN) into a chitosan solution and stirring until homogeneously blended. A 1% aqueous solution of glutaraldehyde was subsequently incorporated into the mixture for crosslinking purposes. The lone pair of electrons on the nitrogen atom of the chitosan amino group (-NH_2_) acts as a nucleophile, attacking the carbonyl carbon atom (-CHO) of the glutaraldehyde aldehyde group, which serves as the electrophilic center, resulting in the formation of an unstable intermediate. This unstable intermediate swiftly eliminates one water molecule, resulting in the formation of a carbon-nitrogen double bond—an imine bond (C=N)—between the nitrogen atom of chitosan and the carbon atom of glutaraldehyde. This bond is also referred to as a Schiff base bond. Every glutaraldehyde molecule has two aldehyde groups [92]. One aldehyde group can interact with an amino group on chitosan chain A, while another aldehyde group can engage with an amino group on chitosan chain B. Numerous glutaraldehyde molecules function as “crosslinking points,” covalently binding the originally separate, linear chitosan polymer chains into a vast, three-dimensional network structure that encompasses the entire system. Enzyme crosslinking constitutes an alternative method of chemical crosslinking. Hydrogels synthesized by enzymatic processes have superior rheological properties, little swelling, and exceptional biocompatibility while also enabling the modulation of toughness, elasticity, and strength by altering the enzyme concentration integrated. Enzyme-crosslinked hydrogels are created using enzyme-catalyzed processes that link polymer chains into a three-dimensional network. Hu et al. developed a silk protein-based, MXene nanoplate-embedded, dual-crosslinked piezoelectric composite hydrogel employing a “dual-crosslinking” approach that integrates enzyme-catalyzed chemical crosslinking with physically induced crystallization. Initially, horseradish peroxidase facilitated the production of covalent di-tyrosine bonds among tyrosine residues in sericin, creating a dynamic initial chemical network. Consequently, ethanol treatment prompted sericin molecules to develop β-fold crystalline structures, acting as physical crosslinking sites to establish a resilient “dual-crosslinked network.” Conductive two-dimensional MXene nanosheets were included into the system before crosslinking. They not only distributed equally across the network to establish conductive channels but also facilitated the self-assembly of sericin on their surfaces, augmenting the stability of the composite material [93].

## 5. Piezocatalysis in Biological Antibacterial Applications

### 5.1. Water Disinfection

**Figure 13:**
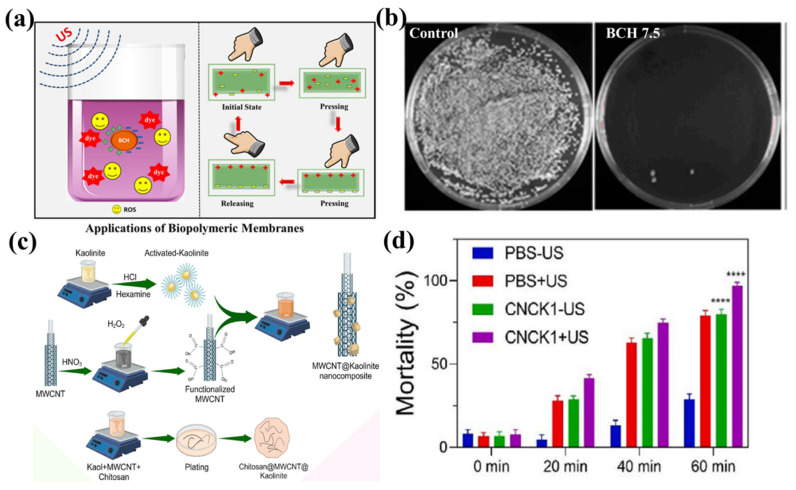
(**a**) Charge distribution in BCH under ultrasonic excitation. (**b**) The Composite Synthesis of Chitosan with Multi-Walled Carbon Nanotubes and Nano-Kaolin Clay [97]. (**c**) Antimicrobial process of piezoelectric biomaterials. (**d**) Agar plate data for the different as prepared samples under US stimulus for 30 min [97], **** *p* < 0.0001. Copyright ©2025, 2024, The Author(s).

Pollution has increasingly intensified due to population expansion and heightened industrial operations. Pathogenic bacteria and parasitic algal blooms result in the degradation of water quality, hence posing direct or indirect risks to human health and impacting fishing yields [94]. Conventional water disinfection techniques are characterized by elevated costs, suboptimal antimicrobial efficacy, and a propensity to induce secondary pollutants, necessitating the exploration of novel disinfection approaches [95]. Piezocatalytic technology demonstrates significant potential in the domain of bio-antimicrobials owing to its high efficiency, straightforward device process, and eco-friendliness, as illustrated in Figure 13a. Natural materials intrinsically exhibit piezoelectric capabilities and provide benefits like biocompatibility, plentiful availability, and cost-effectiveness. When integrated with conventional piezoelectric components, they can lead to an innovative and sustainable method for environmental remediation. Huo et al. effectively integrated the piezoelectric material MoS_2_ into wood and introduced nickel nanoparticles to augment conductivity and create active spots. A wood-derived bulk material was created, employing ultrasonic vibration to activate piezoelectric responses for the breakdown of tetracycline. The piezocatalytic degradation rate of tetracycline attained 95.96% within 60 min [96]. The porous composition of wood serves as an exceptional medium and enhances the surface area in contact with contaminants. MoS_2_ exhibits a non-centrosymmetric crystalline structure that induces charge separation when subjected to mechanical stress. Ni nanoparticles function as conductive conduits and catalysts for enhanced activity. The synergistic interplay among these three components improves electron transport and catalytic activity, resulting in efficient and stable piezocatalytic degradation. Liu et al. fabricated a beryllium ferrite/chitosan membrane composite (BCH) via a straightforward solution casting technique, attaining about 99% elimination of *Staphylococcus aureus* within 30 min following BCH nanoparticle application, as shown in Figure 13b [97]. Under ultrasonic stimulation, BCH experiences lattice deformation, resulting in the formation of macroscopic electric dipoles that facilitate charge carrier separation. This produces superoxide and hydroxyl radicals—reactive species that undermine bacterial cell membrane integrity, impair gene expression, and obstruct electron transport chains, ultimately leading to bacterial cell death. Nevertheless, the piezoelectric coefficients of natural piezoelectric biomaterials are generally modest. To resolve this issue, Li et al. formulated a composite material by integrating the natural piezoelectric biopolymer chitosan (CS) with multi-walled carbon nanotubes and nano-kaolin clay, as seen in Figure 13c. They improved the material’s piezoelectric response by utilizing synergistic effects among the components. Positively charged chitosan strongly adheres to negatively charged natural clay layers via electrostatic interactions and hydrogen bonding. The robust interfacial interaction compels the chitosan chains to organize systematically on the clay surface, augmenting dipole orientation within the composite material and therefore elevating macroscopic polarization. Conversely, the elevated conductivity and surface charge of multi-walled carbon nanotubes produce a strong local electric field within the chitosan matrix. The local electric field more efficiently directs and reorganizes the polar -OH and -NH_2_ groups on the chitosan chains, resulting in enhanced dipole moment variations under mechanical stress. This markedly elevates the piezoelectric coefficient (d_33_) from 3.75 pC/N in pure chitosan to 33.56 pC/N. This composite attained a 97% breakdown rate of pathogenic *E. coli* within 60 min under ultrasonic stimulation, as illustrated in Figure 13d [98].

### 5.2. Biomedical Antimicrobial

**Figure 14:**
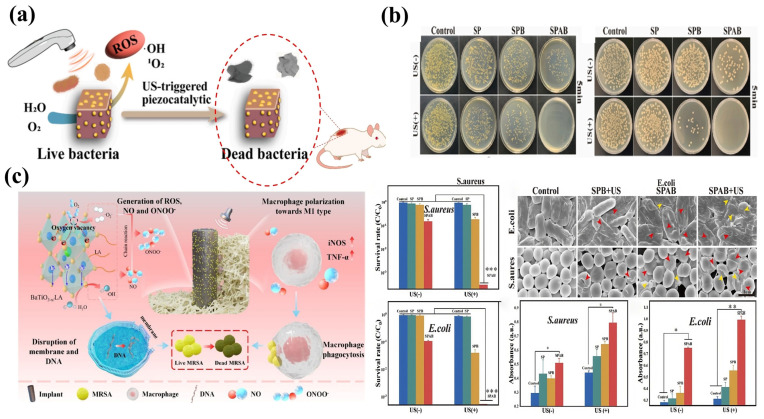
(**a**) Schematic representation of SPAB piezoelectric hydrogel utilization in antibacterial wound healing [102]. (**b**) Mechanisms of antibacterial of BaTiO_3−x_/LA [101]. (**c**) The antibacterial efficacy of SF-MA/PEGDA/Ag@BT hydrogels were evaluated in vitro against *E. coli* and *S. aureus* following a 5 min ultrasound treatment, * *p* < 0.05, ** *p* < 0.01, *** *p* < 0.001. Copyright ©2024, The Author(s).

Bacterial infections represent a significant risk to human health, with five pathogens (*S. aureus*, *E. coli*, *Streptococcus pneumoniae*, *Klebsiella pneumoniae*, and *Pseudomonas aeruginosa*) linked to over 500,000 fatalities each, as per 2019 data [99]. Piezocatalytic technology uses ambient mechanical energy to start reactions, generating ROS that enable long-lasting sterilization and inhibit bacterial growth without creating resistance. This method does away with the necessity for traditional conditions like light oxidants. In recent years, it has gained extensive application in the biomedical antibacterial domain. Piezoelectric bionic bone implants can effectively facilitate antibacterial properties and bone regeneration in the treatment of bone abnormalities. Zhou et al. developed barium/titanium-doped chalcogenide nanomaterials containing oxygen vacancies, achieving bactericidal rates of 93.1% against *S. aureus* and 94.3% against *Escherichia coli* under ultrasound [100]. Moreover, TH-BFBT demonstrated a notable elevation in alkaline phosphatase activity, with the expression levels of bone morphogenetic protein 2 and Runx2 greatly above those of the other groups. The intracellular calcium ion concentration elevated in the TH-BFBT group, suggesting that electrical stimulation facilitated osteogenesis, as shown in Figure 14a,b. In dentistry, piezoelectric implants serve as an effective approach to address implant-associated infections and malintegration in periodontology, dentistry, and orthodontics. Sun et al. developed BaTiO_3_−x/LA composite titanium implants for acoustic-catalyzed synergistic immunotherapy targeting methicillin-resistant *S. aureus* infections [101]. Under ultrasonic irradiation, BaTiO_3_−x nanorod arrays produce ROS, which breakdown LA into NO. NO subsequently combines with ·O_2_^−^ to yield the highly antibacterial peroxynitrite (ONOO^−^). ONOO^−^ is a formidable oxidizing agent with superior antibacterial efficacy compared to ·O_2_^−^. This radical chain reaction boosts the oxidative capacity of radicals and decreases the electron-hole recombination rate by consuming ·O_2_^−^, consequently increasing the therapeutic efficacy of ultrasonic-assisted treatment. The antibacterial efficacy of BaTiO_3_−x/LA attained 97.54%. Novel piezoelectric antimicrobial materials enhance collagen deposition and epithelial remodeling, thereby facilitating wound healing and avoiding skin wound infections. The creation of piezoelectric-based bandages has surfaced as an innovative approach to improve skin wound healing and inhibit bacterial infections. Zhao et al. synthesized SF-MA through the methacrylation modification of sericin (SF) utilizing glycidyl methacrylate (GMA), and subsequently deposited silver (Ag) onto the surface of barium titanate (BT) nanoparticles to produce Ag@BT nanoparticles. Thereafter, SF-MA, Ag@BT, and polyethylene glycol diacrylate (PEGDA) were amalgamated to create a photopolymerizable paste. A unique 3D-printed piezoelectric catalytic SF-MA/PEGDA/Ag@BT (SPAB) hydrogel was created utilizing digital light processing (DLP) technology. The SF matrix within this hydrogel effectively disperses Ag@BT nanoparticles, inhibiting agglomeration and guaranteeing a uniform distribution of piezoelectric catalytic active sites, as illustrated in Figure 14c. The SPAB hydrogel has exceptional piezoelectric catalytic efficacy: following 5 min of in vitro ultrasonic stimulation, it attained bactericidal rates of 99.23% against *E. coli* and 99.96% against *S. aureus* [102].

## 6. Summary and Perspectives

Piezocatalysis stands at the forefront of antimicrobial innovation, offering a sustainable, energy-autonomous paradigm that circumvents the secondary pollution and resistance issues plaguing conventional methods. This review has systematically elucidated the mechano-electrochemical mechanisms underpinning bacterial inactivation, activated by external mechanical stimuli. We have provided a comprehensive survey of the current landscape of piezoelectric biomaterials—ranging from amino acids to complex biopolymers—and consolidated their emerging applications in water purification and biomedical engineering. While bio-based piezocatalysis has demonstrated immense potential, its transition from laboratory research to practical application faces hurdles related to catalytic efficiency, excitation sources, and mechanistic clarity.

To propel this field toward widespread implementation, future research efforts should be directed toward the following critical frontiers:

(1) Rational design of high-performance bio-piezocatalysts: The commercial viability of bio-piezoelectric materials is currently constrained by their modest piezoelectric coefficients compared to inorganic counterparts. Future breakthroughs rely on precise structural modulation. Strategies such as elemental doping, heterojunction engineering, and crystal phase control (e.g., stabilizing the β-phase in glycine) are imperative to amplify the piezoelectric response. Furthermore, the development of novel biodegradable organic-inorganic hybrids that merge the high activity of ceramics with the biocompatibility of organics represents a promising avenue for constructing efficient, eco-friendly antibacterial systems.

(2) Harvesting ubiquitous low-frequency mechanical energy: Current systems predominantly rely on ultrasonic excitation, which restricts applications in scenarios lacking high-frequency sources. A pivotal direction is the development of ultrasensitive catalysts capable of harvesting low-frequency, ambient mechanical energy. Research should pivot towards utilizing hydraulic energy (water flow), triboelectric forces, and, crucially, biomechanical energy (e.g., body movement, respiration, blood flow) to enable self-powered, in vivo sterilization devices.

(3) Synergistic multi-physical field coupling: A solitary piezocatalytic mode often falls short in complex environmental matrices due to rapid ROS quenching or limited penetration depth. Integrating piezocatalysis with other energy-harvesting modalities—such as photocatalysis, pyrocatalysis, or magnetic, thermal fields—is essential. By aligning energy bands and optimizing interface designs, such “tandem” systems can broaden the utilizable energy spectrum and facilitate charge separation, achieving a “1 + 1 > 2” synergistic enhancement in antibacterial efficacy.

(4) Unveiling mechanistic dynamics via advanced characterization: Despite empirical successes, the microscopic mechano-chemical interactions at the bacteria-material interface remain largely a “black box.” There is a pressing need to move beyond standard assays by integrating in situ/operando characterization techniques with advanced theoretical simulations (e.g., Density Functional Theory, Finite Element Analysis). This combined approach will allow researchers to visualize real-time surface charge migration, ROS evolution, and biofilm disruption dynamics, providing a robust theoretical blueprint for targeted material design. In conclusion, advances in material engineering and application strategies are poised to significantly broaden the scope of piezoelectric antimicrobial technology. By effectively harnessing ambient mechanical energy, this bio-based approach offers a compelling, green solution to the global challenges of water pollution and biological contamination.

## Data Availability

No new data were created or analyzed in this study. Data sharing is not applicable to this article.
